# The Veterinarian a Factor in Politics1Read before the Illinois State Veterinary Medical Association, February 21, 1900.

**Published:** 1900-03

**Authors:** T. B. Newby

**Affiliations:** Pana, Illinois


### THE VETERINARIAN A FACTOR IN POLITICS.[1]

By T. B. Newby, V. S.,
PANA, ILLINOIS.

As the present century opens or closes, whichever arm of the argument you wish to accept, it finds us living in an age of ever- and rapidly-changing conditions—an age, indeed, in which the sluggard is remembered only in name; when the successful man keeps abreast of the tide and does not wait for that mighty current to catch him in its drift, until he awakes to activity and views his lost opportunity; a time when the struggle for political supremacy is strained to the last point of human achievements. And he must be both a strategist and tactician to successfully reach the goal. The politician of the old school now rarely attempts an issue by being next to the hearts of the people as a mass. With class arrayed against class, it does not require an Elijah or a Daniel to see that this condition forebodes no good to our nation.

I believe here has been the cause of the veterinary profession wandering around in the wilderness so many years, struggling for recognition ; we were more or less disjointed with the times. We

[1] Read before the Illinois State Veterinary Medical Association, February 21, 1900.

presented just and conservative petitions to the lawmaking bodies, and believed upon their merit, and the men we had elected to office would successfully do the rest.

There were always some men in the profession doing their best and spending their money as well to consummate the attempted issues, while others like a team unable to move their load would give a little pull and stop; and some do much worse at times by trying to block the wheels entirely.

Doubtless oftentimes the executive never knew from personal acquaintance or correspondence that there were any veterinary interests in his district that he was supposed to legislate in favor of; if he did, as often was the case, he failed to render unto Cæsar the things that were Cæsar's.

Particularly is this true of the army bill. As American-born citizens taught to reverence our flag as one of the fundamental principles of manhood from our very birth, emblazoned with our nation's history, emphasized just now when the entire world is looking upon us with wonder and admiration at the valor of American arms in foreign lands and of our navy upon the seas, these inspiring thoughts of loyalty intensify our feelings with odium, chagrin, and mortification, when we remember that the much-needed rank that our profession symbolizes has been ingloriously declined.

Are our aspirations in our proud army to be nothing more than in the dark ages? Is our profession in the army to be relegated far behind that of less successful or chivalrous nations? This is the question for us to settle. After repeated travesties at justice by the United States Congress our efforts have amounted to simply this, viz., a veterinarian appointed under the act of Congress, approved March 2, 1899, is not a commissioned officer or an enlisted man, but a civil employé. Compare this, if you will, with the other armies of the civilized world. In most of them the veterinarian enters the army as a lieutenant, and by promotion may become a veterinary colonel or an intermediate rank. It is estimated in the German army the veterinarian saves twenty times his cost. And doubtless if such an expediency was adopted in the American army and the veterinarian was given like authority in supplying and equipping in common with his other duties, more than this might be saved our government. With this in view it strikes me that the citizen and the tax-payer should be more interested than the veterinarian. The question may be asked, What is our duty as to political matters involving our interests in general? Am I admonishing you to become politicians? No, not in the special sense of its acceptance beyond the incentive of self-preservation. If I was, I should say few if any callings are endowed with so many privileges tributary to influence through extensive acquaintance, and subsequently numerous friends, which he might use to advance political or social ambition if he so elects; but, this is not the objective thought we desire to present. Sentiment based on influence is a vain thing to build on; but rather to be established is the endowment of a profession noble in its calling, so upbuilding in its every duty to society that time cannot mar its inherency.

It is congratulating to know that our profession is rapidly developing this desired condition, and let us not tire in well doing until we take our places as one of the first sciences of the land established in every branch of commerce or concentrated society.

To accomplish this means hard individual work, characterized by thoroughness, dignity, and self-respect, which emphasize and promote character and endow one with reserve intellectual power such as Daniel Webster possessed when he made that instantaneous reply to Robert Y. Hayne, of South Carolina, concerning the doctrine of nullification, in 1830, or the strategy that Dewey possessed when he created that breakfast story while destroying the Spanish fleet in Manilla Bay and wanted to take an inventory of his ammunition.

Legitimate tact judiciously used to impress the thinking people of the best merits of the profession is to be commended. Men of sense never confuse issues or choose the wrong time for their purpose. It is no uncommon thing to see men of high intellectual attainments incapable of self-support, because they lack certain qualifications of imparting their knowledge. Wisdom is useful so far as capable of being applied.

There is to-day vast unhidden possibilities for the ambitious and resourceful veterinarian. Under the plan of the American institutions men rapidly climb the ladder of fame because it is one of the possibilities for all. And that applies to us, and it is our duty to grasp the spirit, develop our latent faculties, and that which belongs to us we will have, because the executive officers and political assemblies cannot then help recognizing us as public benefactors and our social position.

As it now exists it is a political matter, and whatever we receive we must fight for. As in the language of David Harum: there is many a hole in a ten-foot ladder.

